# The G2 checkpoint—a node‐based molecular switch

**DOI:** 10.1002/2211-5463.12206

**Published:** 2017-03-04

**Authors:** Mark C. de Gooijer, Arnout van den Top, Irena Bockaj, Jos H. Beijnen, Thomas Würdinger, Olaf van Tellingen

**Affiliations:** ^1^ Division of Pharmacology/Mouse Cancer ClinicThe Netherlands Cancer InstituteAmsterdamThe Netherlands; ^2^ Department of Pharmacy and PharmacologyThe Netherlands Cancer Institute/Slotervaart HospitalAmsterdamThe Netherlands; ^3^ Division of Drug ToxicologyFaculty of PharmacyUtrecht UniversityThe Netherlands; ^4^ Division of Biomedical AnalysisFaculty of ScienceUtrecht UniversityThe Netherlands; ^5^ Neuro‐oncology Research GroupDepartments of Neurosurgery and Pediatric Oncology/HematologyCancer Center AmsterdamVU University Medical CenterThe Netherlands; ^6^ Molecular Neurogenetics UnitDepartments of Neurology and RadiologyMassachusetts General HospitalBostonMAUSA; ^7^ Neuroscience ProgramHarvard Medical SchoolBostonMAUSA

**Keywords:** CDC25C, cell cycle, CHK1, G2 checkpoint, PLK1, Wee1

## Abstract

Tight regulation of the eukaryotic cell cycle is paramount to ensure genomic integrity throughout life. Cell cycle checkpoints are present in each phase of the cell cycle and prevent cell cycle progression when genomic integrity is compromised. The G2 checkpoint is an intricate signaling network that regulates the progression of G2 to mitosis (M). We propose here a node‐based model of G2 checkpoint regulation, in which the action of the central CDK1–cyclin B1 node is determined by the concerted but opposing activities of the Wee1 and cell division control protein 25C (CDC25C) nodes. Phosphorylation of both Wee1 and CDC25C at specific sites determines their subcellular localization, driving them either toward activity within the nucleus or to the cytoplasm and subsequent ubiquitin‐mediated proteasomal degradation. In turn, this subcellular balance of the Wee1 and CDC25C nodes is directed by the action of the PLK1 and CHK1 nodes via what we have termed the ‘nuclear and cytoplasmic decision states’ of Wee1 and CDC25C. The proposed node‐based model provides an intelligible structure of the complex interactions that govern the decision to delay or continue G2/M progression. The model may also aid in predicting the effects of agents that target these G2 checkpoint nodes.

**Abbreviations**
β‐TrCP: β‐transducin repeat‐containing protein
APC/C: anaphase‐promoting complex or cyclosome
ATM: ataxia telangiectasia‐mutated kinase
ATR: ATM and Rad3‐related kinase
AURKA: aurora kinase A
CAK: cyclin‐activating kinase
CDC14A: cell division control protein 14A
CDC25C: cell division control protein 25C
CDK1: cyclin‐dependent kinase 1
CDS: cytoplasmic decision state
CHK1: checkpoint kinase 1
CK2: casein kinase 2
FBX6: F‐box only protein 6
Hsp90α: heat‐shock protein 90α
KD: kinase domain
MYT1: myelin transcription factor 1
NDS: nuclear decision state
NES: nuclear export signal
PLK1: polo‐like kinase 1
PP1: protein phosphatase 1
PP2A: protein phosphatase 2A
RSK: ribosomal S6 kinase
SCF: Skp1‐Cul1‐Fbox protein
WIP1: wild‐type p53‐induced phosphatase

The eukaryotic cell cycle is tightly regulated and encompasses checkpoints in each of its different phases 1. Cellular checkpoint control is pivotal in minimizing DNA damage accumulation and ensuring genomic integrity during cell cycle progression 2. Thus, not surprisingly, checkpoint deregulation and resulting DNA damage have been implicated in many diseases, including cancer and neurodegenerative disorders 3, 4.

Research conducted during the last two decades supports that nuclear cytoplasmic cycling of important G2 checkpoint proteins – such as cyclin‐dependent kinase 1 (CDK1), Cyclin B1, Wee1 kinase (Wee1), and cell division control protein 25C (CDC25C) – is a key mechanism of G2 checkpoint regulation 5, 6, 7, 8. An elaborate understanding has been established of the various types of protein interactions involved in cellular checkpoint control and the DNA damage response (reviewed in 9). However, a comprehensive spatiotemporal overview of cellular checkpoint dynamics has not yet been reported. Here, we will focus on the human G2 checkpoint as a model checkpoint utilizing the plethora of protein interactions and modifications to regulate nuclear cytoplasmic protein cycling. We identify the diverse post‐translationally modified states of each G2 checkpoint protein undergoing nuclear cytoplasmic cycling. Competing factors determine their state and thereby the subcellular localization and thus the activity of the protein. The outcome of all of these competitions will determine the status of the G2 checkpoint at any given time. Therefore, we propose to call these states the nuclear decision state (NDS) and cytoplasmic decision state (CDS) of a protein. We will describe the G2 checkpoint as a node‐based biomolecular switch in great detail, underlining the importance of various protein interactions and emphasizing subcellular protein localization as a pivotal regulatory factor during checkpoint regulation.

## The nodular basis of checkpoints

**Figure 1:**
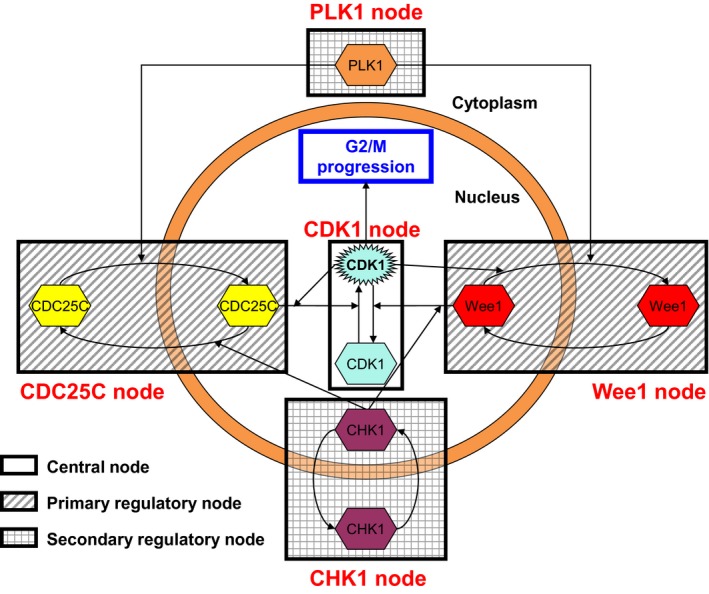
The nodal basis of the G2 checkpoint. The action of the central CDK1‐cyclin B1 node (clear box) is determined by the concerted but opposing activities of the Wee1 and CDC25C primary regulatory nodes (dashed boxes). In their turn, the PLK1 and CHK1 secondary regulatory nodes (gridded boxes) direct the action of the Wee1 and CDC25C nodes by phosphorylation at specific sites that determine their subcellular localization, either driving them toward nuclear accumulation and activity or cytoplasmic localization and subsequent ubiquitin‐mediated proteasomal degradation.

The G1 and G2 checkpoints, although differing in the involvement of specific checkpoint proteins, are in essence node‐based systems revolving around a pivotal CDK node that controls cell cycle progression (Fig. 1). The central CDK2 node regulates the progression to S phase at the G1/S transition, while CDK1 (also known as cell division control protein 2, CDC2) comprises the central checkpoint node of the G2 checkpoint and is responsible for entry into mitosis 4. The central CDK1 node is directly regulated by the primary regulatory Wee1 and CDC25C nodes, which, respectively, phosphorylate and dephosphorylate CDK1 10. In the nucleus, Wee1 phosphorylates CDK1 on Tyr15, inactivating the kinase and thus inducing a G2 arrest, resulting in cell cycle progression inhibition 11, 12. In contrast, the phosphatase CDC25C mirrors Wee1 function by dephosphorylating the inactivating phosphorylation of CDK1 on Tyr15, reactivating CDK1 in the nucleus, and promoting mitotic entry 13, 14.

In turn, the primary regulatory nodes Wee1 and CDC25C are regulated by three regulatory nodes. First, the central CDK1 node itself regulates these nodes. Active CDK1 can phosphorylate both Wee1 and CDC25C resulting in nuclear exclusion of Wee1 and promotion of CDC25C phosphatase activity 14, 15. Via these two activities, CDK1 augments the further formation of active CDK1 through both its regulatory nodes. The result of this mechanism is a truly ingenious molecular switch, where active CDK1, once a certain threshold level is reached, triggers a snowball effect culminating into G2/M progression in a fashion that is irreversible by components of the cellular checkpoint machinery. Obviously, this mechanism calls for meticulous regulation of the CDK1 activation balance.

Secondly, the primary regulatory nodes, Wee1 and CDC25C, are regulated by two secondary regulatory nodes. The polo‐like kinase 1 (PLK1) and checkpoint kinase 1 (CHK1) nodes dictate the cellular localization balance of the Wee1 and CDC25C nodes. PLK1 paves the way for degradation of Wee1 by phosphorylating its Ser53 residue 16. Similarly, the translocation of CDC25C to the nucleus is promoted by phosphorylation on Ser198 by PLK1 17. Taken together, PLK1 promotes G2/M progression through affecting the subcellular localization of both CDK1 regulatory checkpoint nodes. In contrast, CHK1 activity is directly counteractive to that of PLK1 activity. CHK1 promotes the nuclear localization of Wee1 through phosphorylation on Ser642 and prepares CDC25C for cytoplasmic translocation through phosphorylation on Ser216, thus enforcing G2 arrest through both primary regulatory nodes. Below we will discuss in more detail the different nodes and their spatiotemporal role in the G2 checkpoint starting with the secondary regulatory nodes, followed by the primary regulatory nodes and finishing with the central CDK1 node (for a complete overview of all nodes and their spatiotemporal interplay see Fig. S1 and Video S1).

## The PLK1 node

**Figure 2:**
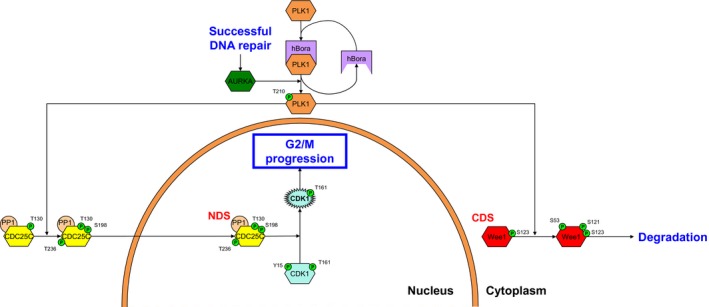
The PLK1 node. Upon successful DNA repair completion, PLK1 is activated by aurora kinase A (AURKA) in cooperation with hBora through phosphorylation of its Threonine‐210 residue. Following activation, PLK1 can promote G2/M progression by driving nuclear accumulation and CDK1 target activity of CDC25C and signaling Wee1 for ubiquitin‐mediated proteasomal degradation.

As a result of various replication events, even in the absence of genotoxic stress, healthy eukaryotic cells acquire basal levels of DNA damage during S phase that may not be resolved until late G2 phase 18, 19, 20, 21. With successful repair of each DNA damage lesion, signaling through PLK1 increases, ultimately resulting in checkpoint recovery and subsequent G2/M progression 22, 23. hBora associates with PLK1, inducing a conformational change between the protein‐binding domain and kinase domain (KD) of PLK1 that exposes the Thr210 site of PLK1 to phosphorylation by active aurora kinase A (AURKA) 24. Activated PLK1 can then phosphorylate CDC25C on Ser198 17, promoting cytoplasmic‐to‐nuclear CDC25C translocation, and Wee1 on Ser53 16, priming Wee1 for ubiquitin‐mediated proteasomal degradation (Fig. 2). Signaling through the secondary regulatory PLK1 node, therefore, is actively driving cells through the G2/M transition by influencing the subcellular distribution of both CDK1 regulatory nodes.

PLK1 has been described as harboring a nuclear translocation signal that allows for tight regulation of its subcellular localization during the cell cycle 25. Already in 1994 Golsteyn *et al*. 26 showed that PLK1 is diffusely localized throughout the cell during interphase. It has been extensively described that PLK1 is involved in the spindle assembly checkpoint during M phase, confirming the importance of active PLK1 at the kinetochore during early mitosis 27, 28, 29. Moreover, a recent study demonstrated that despite being cytoplasmically activated, PLK1 activity is first detected in the nucleus in early G2 phase 30. However, based on current literature it is still conjecture whether nuclear or cytoplasmic PLK1 is predominantly responsible for the role of PLK1 in the G2 checkpoint later in the phase. Here, we propose a model in which cytoplasmic—and not nuclear—PLK1 is the major contributor for its regulatory function in the G2 checkpoint. The effects of PLK1 on nuclear accumulation of CDC25C 17 and Wee1 31—by providing a phosphorylation‐mediated nuclear translocation signal or nuclear stabilization of CDC25C and Wee1—can be explained by both cytoplasmic and nuclear PLK1. However, the finding that myelin transcription factor 1 (MYT1)—a kinase‐targeting CDK1 and further detailed in ‘The CDK1 node’ section described below—is targeted by PLK1 cannot be attributed to a nuclear role of PLK1 in the G2 checkpoint 32. MYT1 is a membrane‐associated Wee1‐like kinase that localizes to the endoplasmatic reticulum and Golgi system 33, 34, and therefore targeting of this kinase by PLK1 has to occur in the cytoplasm. Furthermore, cytoplasmic activity of PLK1 is also required to prime the Cyclin B1‐CDK1 complex for nuclear localization by phosphorylation of serine residues on Cyclin B1 (see also Fig. 6) 35.

## The CHK1 node

**Figure 3:**
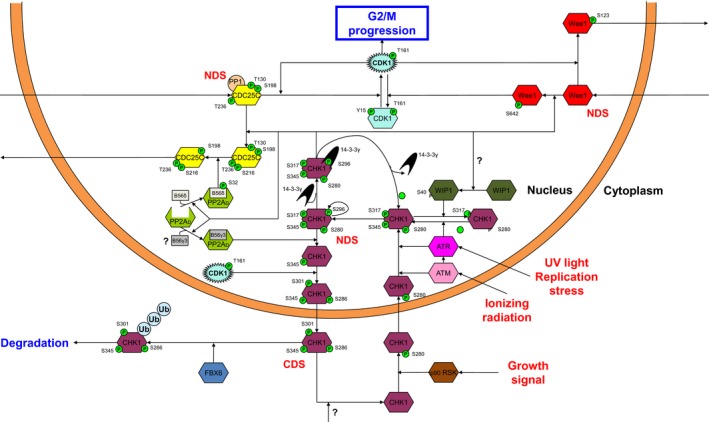
The CHK1 node. CHK1 is translocated to the nucleus by p90 RSK phosphorylation. In the nucleus, CHK1 is activated following DNA damage, either directly by ataxia teleangiectasia‐mutated kinase (ATM), or through its downstream target ATM and Rad3‐related kinase (ATR). The inhibitory activity of CHK1 on G2/M progression is threefold: through activating and nuclearly stabilizing Wee1, through deactivating CDC25C by direct phosphorylation, and through promoting cytoplasmic translocation of CDC25C via activation of PP2A/B56δ. In turn, PP2A/B56γ3 can deactivate CHK1 by dephosphorylation, after which it is primed for nuclear export by CDK1. In the cytoplasm, CHK1 is either targeted for proteasomal degradation by SCF^FBX6^ or reshuttled to the nucleus by dephosphorylation.

Detection of DNA damage during S and G2 phase, causes induction of a G2 checkpoint arrest that allows for proper DNA repair and prevention of mitotic catastrophe 1, 36. The DNA damage signal is relayed to the G2 checkpoint through ataxia telangiectasia‐mutated kinase (ATM), ATM and Rad3‐related kinase (ATR) and CHK1 (Fig. 3). ATM is commonly activated by DNA double strand breaks and is primarily involved in G2 checkpoint arrest 37. ATR is mostly involved in G1 checkpoint arrest and is activated upon DNA Single Strand Break formation 38, 39, but has also been implicated in double strand break repair as a downstream target of ATM 40. Following growth signaling through the mitogen‐activated protein kinase pathway, p90 ribosomal S6 kinase (RSK) phosphorylates CHK1 at the Ser280 residue, promoting its nuclear localization 41. In the nucleus, both ATM 42, 43 and ATR 44 can phosphorylate their downstream target CHK1 on the Ser317 and Ser345 amino acid residues, inducing CHK1 autophosphorylation on Ser296 45, 46 and enabling CHK1 to carry out its role in inducing G2 checkpoint arrest 47. In contrast, Ser40‐activated wild‐type p53‐induced phosphatase (WIP1) can prevent CHK1 autophosphorylation by dephosphorylating the Ser345 residue 48, a process that might be autoregulated by a feedback loop similar to that proposed for CHK2 49, 50. The autophosporylated form of CHK1 can best be designated as the CHK1 NDS, since it is competed for by 14‐3‐3 γ and protein phosphatase 2A (PP2A). Ser296 phosphorylation favors nuclear activity and can be stabilized by association with 14‐3‐3 γ 51. In contrast, exhibition of CHK1 activity consumes a phosphorylated residue. We speculate that the phosphorylated Ser296 residue of CHK1 is consumed, thereby inducing dissociation of 14‐3‐3 γ and again allowing CHK1 to autophosphorylate or be targeted by WIP1.

CHK1 controls both CDK1 regulatory nodes by managing the subcellular localization balance of Wee1 as well as CDC25C. CHK1 phosphorylates Wee1 on Ser642, preventing it from being targeted for extranuclear translocation and activating its CDK1‐directed kinase activity 52, 53. Conversely, CHK1 phosphorylates the Ser216 residue of CDC25C, signaling it for extranuclear translocation and preventing activation of its phosphatase activity by CDK1 54. Moreover, CHK1 also facilitates the second step in extranuclear translocation signaling of CDC25C through its effect on PP2A. PP2A is a trimeric dual‐specific phosphatase that always consists of a structural (A), catalytic (C), and regulatory subunit (B) 55. As the diversity in A and C isoforms is limited but a range of highly diverse B isoforms exist with different subcellular localizations, substrate recognition of the PP2A complex is generally determined by the regulatory B isoform associated with the AC dimer (PP2AD) 56. On one hand, CHK1 can phosphorylate the nuclear B56δ regulatory PP2A subunit on the Ser32 residue, promoting the association of the PP2AD/B56δ complex 57. Subsequently, the PP2AD/B56δ complex can dephosphorylate CDC25C on Thr130 (which is phosphorylated by CDK2, further addressed in ‘The CDC25C node’ below), further promoting cytoplasmic translocation of CDC25C. Interestingly, a recent study suggests that Greatwall kinase promotes nuclear CDK1 activity following DNA damage recovery through inhibition of the PP2A complex, specifically in promoting dephosphorylation of CDK1Tyr15 in the nucleus 58. Since Greatwall is known to inhibit PP2A, we speculate that Greatwall and CHK1 may potentially have antagonistic effects on PP2A complex activity in the context of DNA damage. On the other hand, a negative feedback loop exists between CHK1 and PP2A where CHK1 can promote the association of a nuclear B regulatory subunit with the PP2A dimer 59. The PP2AD/B trimer can then dephosphorylate the CHK1 NDS, rendering it inactive and compromising G2 arrest 60. Although the specific PP2A regulatory subunit responsible for dephosphorylation of CHK1 is still unknown, PP2AD/B56γ3 has been described to dephosphorylate checkpoint kinase 2 (CHK2) 61. Since CHK1 and CHK2 share many downstream targets, encompass similar KDs, and are both inhibited by AZD7762, the nuclearly localized B56γ3 subunit is a likely candidate to exhibit affinity toward both CHK1 and CHK2 39, 62, 63. Thus, PP2AD/B56δ enables cytoplasmic translocation of CDC25C and activates G2 arrest, whereas PP2AD/B56γ3 may prevent CDC25C translocation to the cytoplasm and antagonizes G2 arrest. Importantly, the opposite functions of these two different PP2A complexes downstream of CHK1 may help to explain the recent observation that inhibition of the catalytic subunit of PP2A by okadaic acid resulted in the attenuation of G2 arrest while increasing phosphorylated CHK1 levels 64.

Following dephosphorylation by PP2A, CHK1 can be targeted by the CDK1‐Cyclin B1 complex, reinforcing nuclear CDK1 activity. This interaction has been shown to promote Crm‐1 (exportin‐1)‐mediated translocation of CHK1 to the cytoplasm by phosphorylation of the Ser286 and Ser301 residues 65. Interestingly, a reciprocal cytoplasmic interaction also been described for CHK1 in regulating Cyclin B1‐CDK1 localization to the centrosomes, thereby preventing premature mitosis 66. In the cytoplasm, CHK1^S268/S301/S345^ is competed for by the E3 ubiquitin ligase Skp1‐Cul1‐Fbox F‐box only protein 6 (SCF^FBX6^) on one hand and phosphatases on the other hand, designating this form as the CDS. FBX6 targets CHK1 for ubiquitin‐mediated proteasomal degradation by phosphodegron recognition of the Ser345 residue 67, whereas phosphatase activity would prime CHK1 for re‐entry into the nucleus. The identity of these phosphatases is not established yet but prime candidates include PP2A, WIP1, and protein phosphatase 1 (PP1).

## The Wee1 node

**Figure 4:**
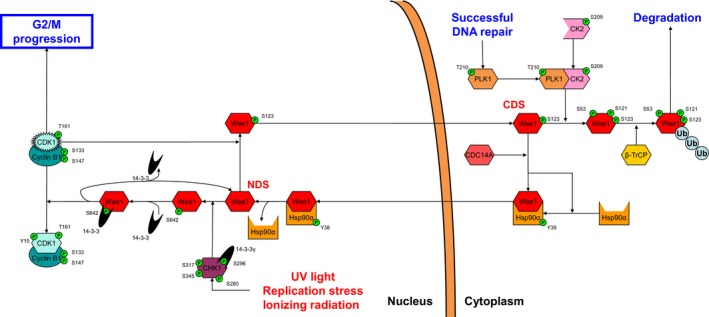
The Wee1 node. Wee1 undergoes nuclear cytoplasmic cycling that is important for determining its inhibitory effect on G2/M progression through the CDK1 node. Wee1 is shuttled into the nucleus by heat‐shock protein 90α (Hsp90α), where it reaches its NDS. Phosphorylation of the Wee1 NDS by CHK1 promotes CDK1‐directed activity of Wee1, while phosphorylation by CDK1 promotes translocation back to the cytoplasm. In the cytoplasm, Wee1 reaches its CDS that is either primed for ubiquitin‐mediated proteasomal degradation by phosphorylation by the PLK1‐CK2 complex or prepared for re‐entry into the nucleus by dephosphorylation by CDC14A.

Wee1 kinase is the primary negative regulator of the CDK1 node through phosphorylation of active CDK1 on the Tyr15 residue (Fig. 4) 68, 69. Wee1 has been shown to be differentially localized throughout the cell cycle 70. Following its synthesis in the cytoplasm, Wee1 can be shuttled into the nucleus by the phosphorylated chaperone protein heat‐shock protein 90α (Hsp90α) 71, 72. Moreover, complex formation with Hsp90α stabilizes cytoplasmic Wee1 by preventing it from degradation 73. Interestingly, Wee1 facilitates its own nuclear translocation by phosphorylation of the Tyr38 residue of Hsp90α 74.

Once transported into the nucleus, Wee1 needs to be released from Hsp90α to carry out its downstream function, which occurs by a mechanism that is not yet elucidated but might involve dephosphorylation of Hsp90α by any tyrosine phosphatase. This unbound form of Wee1 can best be designated as the Wee1 NDS since two kinases compete for this unphosphorylated Wee1. On one hand CHK1 targets the Ser642 residue of Wee1, favoring the Wee1 decision toward phosphorylation of CDK1 53. On the other hand, the active Cyclin B1‐CDK1 complex targets the Ser123 residue, which favors the fate of the Wee1 decision toward extranuclear translocation 15.

Phosphorylation of the Ser642 residue by CHK1 creates a binding site for the cup‐shaped phosphorylation stabilization family of 14‐3‐3 proteins, which shelters the phosphorylated residue and stabilizes phosphorylated Wee1^S642^ 53. This configuration of Wee1 is the active form that can phosphorylate CDK1 on the Tyr15, maintaining the cyclin B1‐CDK complex in the inactive state and preventing G2/M progression. As recently described in yeast, Cks complex formation possibly mediates this targeting of CDK1 by Wee1 by facilitating protein association 75. Since active Wee1 only harbors one phosphorylated residue, we speculate that Wee1‐mediated Tyr15 phosphorylation of CDK1 goes at the expense of the phosphorylated Ser642 residue causing release of 14‐3‐3, thereby returning Wee1 to the Wee1 NDS. In contrast, phosphorylation of the Wee1 NDS by CDK1 generates a signal for cytoplasmic translocation of Wee1 that also acts as a phosphodegron once Wee1 translocation has been completed 16, 76. Although the mechanism by which translocation is mediated is unclear, one might speculate about the involvement of heat‐shock proteins since these important cellular chaperone proteins have also been shown to transport Wee1 into the nucleus.

The resulting cytoplasmic phosphorylated Wee1^S123^ can be designated as the Wee1 CDS since, again, two proteins compete for this form. At one end, cell division control protein 14A (CDC14A) dephosphorylates Ser123, undoing the action of CDK1, preventing Wee1 degradation, and completing the Wee1 cycle by again enabling complex formation with Hsp90α 77. At the other end, active phosphorylated PLK1^Y210^ in a complex with phosphorylated casein kinase 2 (CK2) competes with CDC14A for the Wee1 CDS. Phosphorylation of the Ser53 residue by PLK1 and the Ser121 residue by CK2 creates two additional phosphodegrons 31, 78, 79. Ultimately, the three phosphodegrons generated by CDK1, CK2, and PLK1 fiercely promote docking of the E3 ubiquitin ligase Skp1‐Cul1‐Fbox β‐transducin repeat‐containing protein (SCF^β‐TrCP^), preparing Wee1 for proteasome‐mediated degradation 16. This degradation most likely occurs in the cytoplasm, since the initial phosphodegron that CDK1 generates on Wee1 also acts as a cytoplasmic localization signal.

## The CDC25C node

**Figure 5:**
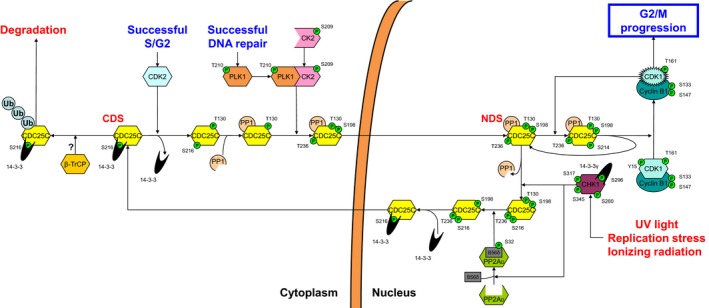
The CDC25C node. The CDC25C node is characterized by nuclear cytoplasmic cycling that regulates its effect on promoting G2/M progression. Following successful completion of S and G2 phase, CDK2 primes CDC25C for nuclear translocation that is subsequently facilitated by PP1 and the PLK1‐CK2 complex. When it reaches the NDS, targeting by activated CDK1 stimulates activity of CDC25C toward the CDK1‐Cyclin B1 complex while CHK1 drives cytoplasmic translocation both directly and through PP2A/B56δ. Back into the cytoplasm, at the CDS, CDC25C can either be primed for re‐entry into the nucleus by CDK2 or targeted for ubiquitin‐mediated proteasomal degradation.

In human cells, three isoforms of CDC25 have been identified that can in part not only compensate for each other's role upon perturbation of the cell cycle machinery but also perform distinct functions throughout the cycle under physiological conditions 80, 81, 82. While CDC25A and CDC25B instigate Cyclin B1‐CDK1 activation, CDC25C is responsible for stimulating and maintaining the full‐blown Cyclin B1‐CDK1 activation that ultimately determines to pass the G2 checkpoint 13. It is this role that makes CDC25C an essential node in regulating the decision of the G2 checkpoint. The CDC25C node mirrors many features of the Wee1 node, including a nuclear cytoplasmic cycle, the presence of NDS and CDS, and an important regulatory role of proteasomal degradation (Fig. 5). We here propose that nuclear translocation of CDC25C, ultimately promoting G2/M progression, is under the control of cytoplasmic CDK2, the CDK responsible for the G1/S and S/G2 transitions in complex with Cyclin E and Cyclin A, respectively 4. Several findings support this hypothesis. First, as a result of successful S phase completion, cellular levels of Cyclin A will increase during G2 phase, indicating that high Cyclin A‐CDK2 levels are correlated with onset of mitosis 7. Secondly, it has been demonstrated that Cyclin A‐CDK2 complexes rapidly shuttle between the cytoplasm and the nucleus, allowing cytoplasmic targeting of CDC25C by CDK2 8. Thirdly, active CDK2 can phosphorylate CDC25C on the Thr130 residue, signaling CDC25C for nuclear translocation and initiating the G2/M transition 83, 84. In turn, this phosphorylation has been shown to cause release of 14‐3‐3, which shields the phosphorylated Ser216 residue of CDC25C at the CDS of CDC25C, allowing weak association with PP1—a serine/threonine phosphatase—and subsequent Ser216 dephosphorylation. Since 14‐3‐3‐bound CDC25C is rapidly translocated to the cytoplasm, we speculate that such a role for CDK2 can therefore only be cytoplasmically localized 85.

PP1 remains associated with CDC25C during its nuclear translocation, facilitating its own nuclear shuttling 86. Dephosphorylation of Ser216 allows dual phosphorylation of CDC25C by a complex consisting of PLK1 and CK2. Activated PLK1 can phosphorylate CDC25C on Ser198. Since this residue is located within the nuclear export signal (NES), PLK1 thereby ultimately promotes nuclear retention by preventing subsequent nuclear exclusion 17. Moreover, active CK2 phosphorylates the Thr236 residue of CDC25C, creating a nuclear localization signal and mediating binding of the importin‐α/β complex that subsequently shuttles CDC25C to the nucleus 87.

Nuclear triple phosphorylated CDC25C^T130/S198/T236^ that is weakly associated with PP1 can be designated as the NDS of CDC25C, since both CDK1 and CHK1 compete for phosphorylation to determine its fate. Favoring entry into mitosis, CDK1 can phosphorylate the Ser214 residue as part of a positive feedback loop, enabling CDC25C to carry out its phosphatase function on CDK1 14, 88. Moreover, phosphorylation of Ser214 strengthens association with PP1, further stabilizing the active form of CDC25C 86. The activating dephosphorylation of its target CDK1 most likely consumes the phosphorylated Ser214 residue of CDC25C, since no phosphatase has been described to target this residue and dephosphorylation of any of the other residues would result in rapid translocation to the cytoplasm. The consumption of the Ser214 residue of CDC25C again weakens the association with PP1 and effectively returns CDC25C to the NDS of CDC25C.

Opposite to the action of CDK1, CHK1 can phosphorylate the NDS of CDC25C on the Ser216 residue, favoring nuclear exclusion and causing dissociation of PP1 54, 86. Moreover, active CHK1 causes active PP2AD/B56δ complex formation and subsequent dephosphorylation of the Thr130 residue of CDC25C 57. Subsequently, 14‐3‐3 binding stabilizes the phosphorylated Ser216 residue and promotes removal of the phosphorylated Ser198 residue through induction of a conformational change 54. Since this causes the nuclear exclusion signal to become unphosphorylated again, 14‐3‐3 binding induces cytoplasmic translocation of CDC25C 85, 89. The cytoplasmically localized, 14‐3‐3 bound, phosphorylated CDC25C can be designated as the CDS of CDC25C, since CDK2 competes for this state with the cellular degradation machinery to again initiate nuclear translocation of CDC25C.

Cytoplasmically located phosphorylated CDC25C^S216^ is recognized by components of the degradation pathways, promoting G2 arrest and implicating the phosphorylated S216 residue as a phosphodegron 90. In contrast to Wee1, the precise players involved in CDC25C degradation are still unclear. However, several observations argue in favor of CDC25C and Wee1 following identical routes of degradation. First, experiments with Arsenite have confirmed that ubiquitin‐mediated proteasomal degradation is responsible for CDC25C degradation 91. Secondly, CDC25A, the homolog of CDC25C that is predominantly active in the G1/S transition, has been reported to be ubiquitinated by the E3‐ligase SCF^β‐TrCP^ 92, although a role for the Anaphase‐Promoting Complex or Cyclosome (APC/C) has also been described 93.

## The CDK1 node

**Figure 6:**
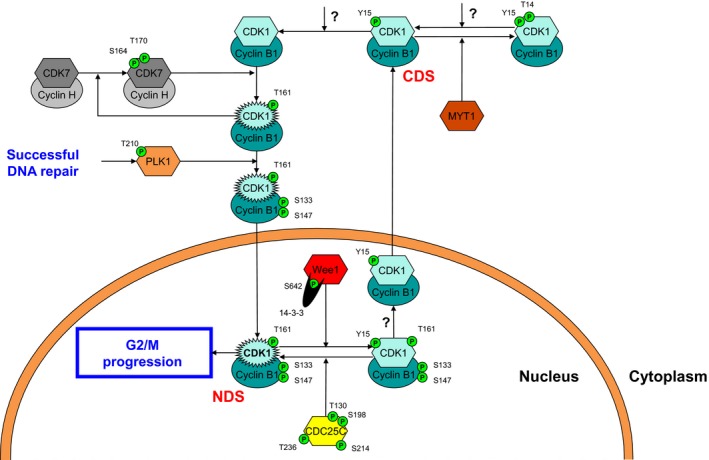
The CDK1 node. Nuclear cytoplasmic cycling regulates CDK1 activity. The CDK1‐Cyclin B1 is cytoplasmically activated by the CDK7‐Cyclin H complex and subsequently primed for nuclear translocation by PLK1. In the nucleus, the NDS of CDK1 can be deactivated by Wee1‐mediated phosphorylation, a process that is counteracted by CDC25C‐mediated dephosphorylation. Following Wee1‐mediated inactivation, the CDK1‐Cyclin B1 complex is primed for cytoplasmic translocation, most likely through dephosphorylation of Cyclin B1, where it reaches its CDS. Here, targeting by MYT1 further inactivates CDK1 while phosphatase activity enables re‐entry into the nucleus.

Even though CDK1 can form a complex with other cyclins earlier in the cell cycle, the Cyclin B1‐CDK1 complex is responsible for triggering mitotic onset. The Cyclin B1‐CDK1 complex is localized cytoplasmically during interphase but is rapidly translocated to the nucleus to instigate G2/M transition during prophase 7, 94. CDK1 is activated in the cytoplasm by cyclin‐activating kinase (CAK) through phosphorylation of its Thr161 moiety (Fig. 6), prior to its nuclear translocation 13, 95. CAK is a kinase complex comprised of CDK7 and Cyclin H and is regulated by a positive feedback loop through Cyclin B1‐CDK1 96, 97. This complex activates CAK by phosphorylating the Ser164 and Thr170 residues of CDK7, fortifying its own activation 98, 99. The activated Cyclin B1‐CDK1 complex is recognized by PLK1, which was originally thought to signal the complex for importin‐β‐mediated nuclear uptake through phosphorylation of the Ser133 and Ser147 residues of Cyclin B1 8, 100, 101. Later, it was speculated that phosphorylation of the complex by PLK1 prevents subsequent nuclear exclusion since both residues are located within the Cyclin B1 NES 8, 35, 102, although the targeting of the Ser133 residue by PLK1 remained controversial 103. Complex formation of PLK1 with CK2 might offer an explanation to resolve this controversy, since the Ser133 residue of Cyclin B might be targeted by CK2, whereas the Ser147 of Cyclin B is targeted by PLK1. First, since the PLK1‐mediated regulation of subcellular localization of CDC25C (Fig. 5) and Wee1 (Fig. 4) is carried out in complex with CK2, a similar PLK1 mechanism in conjunction with CK2 is not unlikely to be also involved in the cytoplasmic‐to‐nuclear transport regulation of Cyclin B1‐CDK1. Moreover, the generation of two phosphorylated residues on Cyclin B1 adds to the likelihood of two kinases being involved. More recently, important work has provided important new information on the controversy surrounding the role of PLK1, and possibly CK2, in promoting nuclear localization of Cyclin B1‐CDK1, by demonstrating that nuclear Cyclin B1‐CDK1 itself promotes the increased nuclear entry of cytoplasmic Cyclin B1‐CDK1, although the precise mechanism by which this occurs has not yet been elucidated 104. Interestingly, the observation that phosphorylated Cyclin B1 allows the Cyclin B1‐CDK1 complex to stably bind to mitotic chromatin could suggest that Cyclin B1 phosphorylation by PLK1, and possibly CK2, is important for the maintenance of the nuclear Cyclin B1‐CDK1 fraction 105.

In the nucleus, the active Cyclin B1‐CDK1 can be designated as the NDS of Cyclin B1‐CDK1, since Wee1 competes for this state with the mitosis‐promoting activity of CDK1. Moreover, the active Cyclin B1‐CDK1 complex directly counteracts the effects of CHK1 in the nucleus by competing for the NDS of Wee1 and CDC25C and priming CHK1 for nuclear export (Fig. 3). Wee1 phosphorylates the NDS of Cyclin B1‐CDK1 on the Tyr15 residue of CDK1, a modification counteracted through dephosphorylation by CDC25C. Tyr15 phosphorylation inactivates CDK1 and signals it for Crm‐1 (exportin‐1)‐mediated nuclear exclusion 8, 106, 107. Thus far, the mechanism responsible for cytoplasmic translocation has not been identified, but the requirement for dephosphorylation of the Cyclin B1 NES points toward abundant nuclear phosphatases such as PP2A and PP1 as likely candidates responsible for this process 108.

In the cytoplasm, MYT1 competes with a yet unidentified phosphatase for the phosphorylated CDS of Cyclin B1‐CDK1^Y15^. MYT1 further consolidates the inactivation of CDK1 by phosphorylating the Thr14 residue of CDK1, sequestering it from the nuclear cytoplasmic cycle 109. Such a cytoplasmic role for MYT1 explains the observation that it is not essential for cell cycle arrest under normal conditions with low levels of DNA damage, but MYT1 does strengthen the Wee1‐induced G2 checkpoint arrest through its effects on CDK1 after extensive DNA damage 110.

Indeed, it was shown that overproduction of MYT1 sequesters CDK1 in the cytoplasm by preventing nuclear import 109. Whether the phosphorylated Thr14 residue may act as a phosphodegron is not yet clear, but the observation that CDK1 levels are unchanged following overexpression of MYT1 suggests against a role for MYT1 in ubiquitin‐mediated proteasomal degradation of CDK1. Rather, Cyclin B1‐CDK1 regulates Cyclin B1 degradation in late mitosis through phosphorylation of APC/C 108, 111, 112. Alternatively, the sequestered Cyclin B1‐CDK1^T14/Y15^ might be reintroduced into the nuclear cytoplasmic cycle and subsequently further dephosphorylated by a yet unidentified phosphatase, allowing reactivation by CDK1 by CAK 113. The ability of CDC14 to dephosphorylate CDK1 upon mitotic exit together with its cytoplasmic localization during the G2 phase makes it a potential candidate for this action, although both PP1 and PP2A have also emerged as likely candidates for CDK1 dephosphorylation 77, 108.

## Implications of the node‐based model

**Figure 7:**
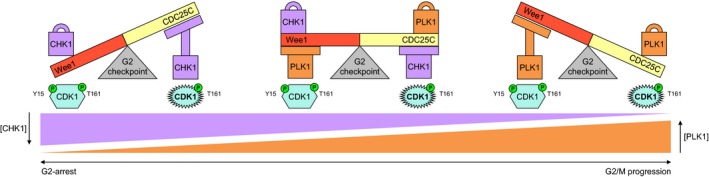
Functional implications of the G2 checkpoint balance. The G2 checkpoint can be considered as a scale, where both arms are represented by the primary regulatory Wee1 and CDC25C nodes that pivot around the central CDK1 node and tip toward either active or inactive CDK1. The arms of the scale are in turn balanced by the secondary regulatory CHK1 and PLK1 nodes that act as either weights or hydraulic pumps. Following completion of S and G2 phase, high activity of the CHK1 node tips the scale in favor of inactive CDK1. With increasing DNA repair, CHK1 node activity gradually diminishes while PLK1 node activity complementarily increases. This shift first levels the balances and ultimately tips the G2 checkpoint in favor of CDK1 activity and G2/M progression.

The node‐based model of the G2 checkpoint represents a tightly controlled biomolecular switch. The Wee1 and CDC25C nodes are two arms of a scale that is influenced by the CHK1 and PLK1 nodes (depicted as weights and hydraulic arms) to tip to either the active or inactive form of CDK1 (Fig. 7). Even under normal growth conditions, cells will always be halted upon arrival at the G2 checkpoint because of high CHK1 activity as a result of replication stress. This arrest allows for DNA repair. With successful repair of each DNA damage lesion PLK1 levels will rise, continuously bringing more balance to the scale. Once PLK1 activity exceeds CHK1 activity, the balance is tipped toward G2/M progression and feedback loops between CDK1 and CDC25C, Wee1, and CHK1 ensure that the balance cannot be restored and essentially becomes an irreversible biomolecular switch. In line with this, recent work demonstrated that G2 checkpoint recovery is dependent on a PLK1 activity threshold and can occur in the presence of a range of residual DNA damage signaling, resulting in heterogeneity in checkpoint fidelity 23.

**Figure 8:**
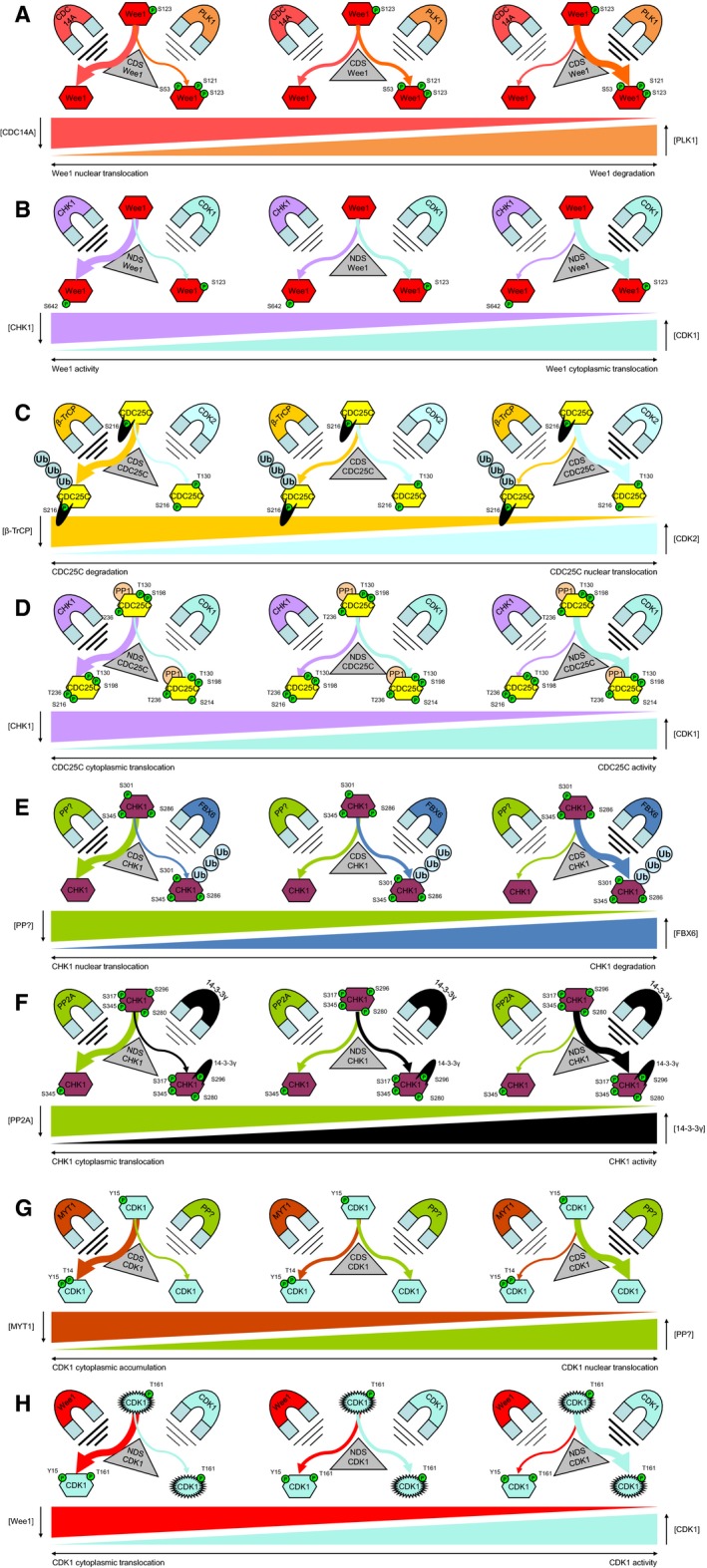
Functional outcome of the decision states involved in the G2 checkpoint. The NDS and CDS can be seen as conformations of a protein that are targeted by competing factors that determine the functional effect of a node on G2 checkpoint. The competing factors can be regarded as magnets that pull with varying strength, thereby routing the protein to a specific conformation. Subsequently are depicted the (A) Wee1 CDS, (B) Wee1 NDS, (C) CDC25C CDS, (D) CDC25C NDS, (E) CHK1 CDS, (F) CHK1 NDS, (G) CDK1 CDS, and (H) CDK1 NDS.

Although not yet all details of the G2 checkpoint have been resolved, the model of the G2 checkpoint as proposed here already offers support in explaining some of the thus far poorly understood observations in studies using inhibitors interfering in the checkpoint. The interplay between the different G2 checkpoint nodes is predominantly determined by influencing the decision states of Wee1, CDC25C, and CDK1 (Fig. 8). Consequently, inhibition of key proteins influencing certain decision states might result in subcellular accumulation or, alternatively, changes in the level of degradation of the target.

For instance, as has been reported, inhibition of CHK1 would result in enhanced nuclear‐to‐cytoplasmic export and subsequent degradation of Wee1 since its competition with CDK1 is compromised. On the other hand, however, inhibition of the KD of Wee1 by a small molecule would not affect the decision states and therefore does not change cellular Wee1 levels 114. The effect of CHK1 inhibition on CDC25C would be exactly opposite. Since CHK1 normally affects the CDC25C NDS by signaling for nuclear‐to‐cytoplasmic export, inhibition of CHK1 using small molecules would result in nuclear accumulation of CDC25C and thus increased cellular levels as a result of decreased degradation. This has been reported for its homolog CDC25A and could thus likewise be true for CDC25C 62, 115, 116. Moreover, the proposed nuclear cytoplasmic cycling of CHK1 implies that inhibition of nuclear localized CHK1 results in nuclear accumulation and therefore prevention of CHK1 degradation, thus resulting in increased cellular CHK1 levels, as has been observed 117. Inhibition of PLK1 would result in cytoplasmic accumulation of CDC25C since nuclear uptake of CDC25C is inhibited while being unavailable for degradation, as has been demonstrated using antisense mRNAs 118. As a final example, PLK1 inhibition would also result in increased Wee1 protein levels since degradation of Wee1 is attenuated at the CDS of Wee1. Together, the proposed model allows for the accurate prediction of the effects of interfering in the G2 checkpoint, making it a highly informative tool for the development of therapies focused on interfering in the G2 checkpoint.

## Conclusions and future directions

Although the last two decades have generated a framework of the biomolecular network of the G2 checkpoint, many interesting questions remain. Several steps of the G2 network remain to be elucidated in full detail, among which the factor responsible for the cytoplasmic phosphatase activity on Cyclin B1‐CDK1, the signal priming Cyclin B1‐CDK1 for extranuclear transport, the E3‐ligase responsible for CDC25C degradation, and the specific PP2A regulatory subunit determining CHK1 substrate recognition. Moreover, the cytoplasmic activation of the Cyclin B1‐CDK1 complex has interesting implications for the regulatory effect of CDK1 on the Wee1 and CDC25C nodes, although the extent to which these implied mode of actions truly influence the nodal balance of the G2 checkpoint has yet to be determined. On the one hand, cytoplasmically active CDK1 might already activate CDC25C for CDK1‐targeted activity prior to nuclear entry, possibly preventing nuclear phosphorylation by CHK1 and thereby loosening the control of CHK1 on the NDS of CDC25C. On the other hand, it might directly counteract the effect of the phosphatase CDC14A on cytoplasmic Wee1, promoting Wee1 to re‐enter the NDS of Wee1 by phosphorylating it and subsequently promoting degradation of Wee1. Together, this would allow CDK1 to simultaneously promote G2/M progression in the nucleus and the cytoplasm, adding to the strength of the G2 molecular switch.

Although beyond the scope of this review, it is intriguing to note that, many reports suggest a role for several G2 checkpoint players or homologs thereof in the regulation of the G1 or intra‐S checkpoints. Among these are Cyclin A, CDK2, CDC25A, CAK, PP2A, PP1, CHK1/2, PLK2/3, and Wee1 39, 119, 120, 121, 122, 123, 124. This plethora of similarities to the G2 checkpoint therefore suggests that the G1 checkpoint is comprised of a similar nodal system with nuclear cytoplasmic cycling, decision states, and important degradation steps. Interesting questions are therefore raised about the similarities and differences between the G1 and G2 checkpoints and their implications for the effect of G2 checkpoint interference on the G1 checkpoint.

In summary, the G2 checkpoint is an ingenious node‐based molecular switch which outcome is determined by the interplay of the PLK1, CHK1, Wee1, CDC25C and CDK1 nodes that are influenced by DNA damage and repair signaling. Together, this system allows the cell to intricately relay DNA status information to the cell cycle machinery, making it a pivotal process in maintaining cellular integrity.

## Author contributions

MG, AT, and IB searched the literature; MG, AT, IB, and OT designed the model; MG, OT, JB, and TW wrote the manuscript.

## Data Accessibility

## Supporting information


**Fig. S1.** Complete overview of the G2 checkpoint network.

**Video S1.** The G2 checkpoint ‐ a node‐based molecular switch.
