# Supplementary material for: The G2 checkpoint—a node‐based molecular switch
Source: FEBS Open Bio. 2017 Mar 4;7(4):439–55. doi: 10.1002/2211-5463.12206 (PMC5377395; doi:10.1002/2211-5463.12206)
Supplement: Supplementary file 1 — Fig. S1. Complete overview of the G2 checkpoint network. [file FEB4-7-439-s001.ppt]

## Slide 1
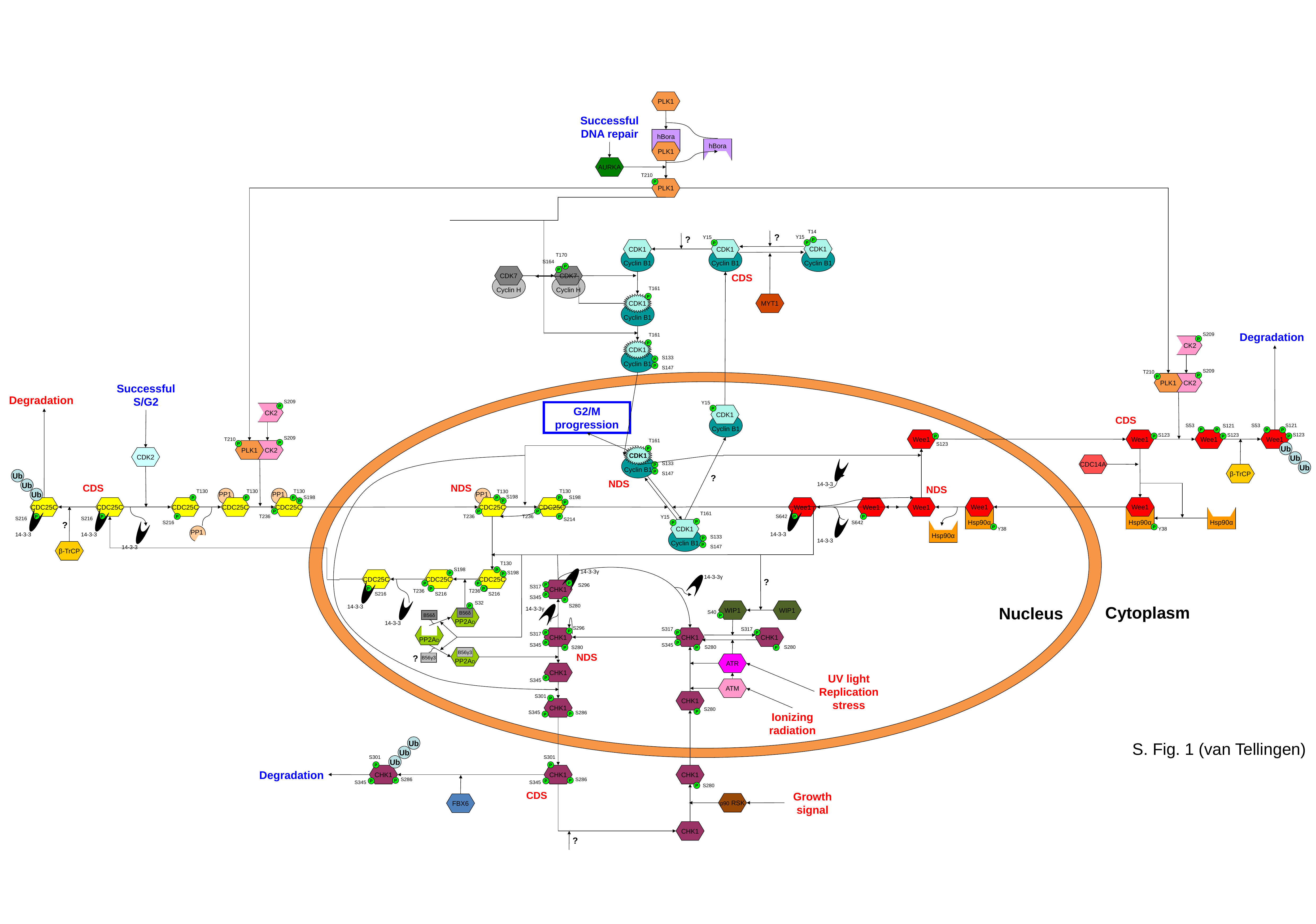

PLK1
Successful DNA repair
hBora
hBora
PLK1
AURKA
T210
PLK1
P
T14
?
?
Y15
Y15
P
CDK1
P
CDK1
CDK1
P
Cyclin B1
Cyclin B1
Cyclin B1
T170
S164
P
CDK7
CDK7
P
CDS
Cyclin H
Cyclin H
T161
P
CDK1
MYT1
Cyclin B1
Degradation
S209
T161
P
 CK2
P
CDK1
Cyclin B1
S133
P
P
S147
S209
T210
P
P
PLK1
 CK2
Successful S/G2
Degradation
S209
Y15
G2/M progression
P
 CK2
P
CDK1
CDS
Cyclin B1
S53
S53
S121
S121
P
P
P
P
S123
Wee1
S123
S123
Wee1
Wee1
Wee1
S209
P
P
P
P
T210
T161
S123
P
P
PLK1
 CK2
Ub
P
CDK1
CDK2
Ub
CDC14A
Cyclin B1
S133
Ub
P
β-TrCP
P
S147
Ub
?
NDS
14-3-3
Ub
CDS
NDS
NDS
T130
T130
T130
T130
T130
Ub
PP1
PP1
PP1
S198
S198
S198
P
P
P
P
P
Wee1
Wee1
CDC25C
CDC25C
CDC25C
CDC25C
CDC25C
CDC25C
CDC25C
Wee1
Wee1
Wee1
P
P
P
Hsp90α
Hsp90α
Hsp90α
P
P
P
T161
T236
T236
T236
S642
Y15
S216
P
S216
P
P
P
P
P
S214
?
S216
S642
P
P
CDK1
Hsp90α
P
Y38
P
Y38
PP1
Cyclin B1
14-3-3
14-3-3
14-3-3
S133
P
14-3-3
β-TrCP
14-3-3
P
S147
T130
S198
14-3-3γ
P
S198
CDC25C
CDC25C
P
CDC25C
P
14-3-3γ
?
CHK1
P
S296
P
P
S317
P
P
P
P
P
T236
T236
S216
S216
S216
P
S345
P
S32
Cytoplasm
S280
WIP1
WIP1
Nucleus
14-3-3
P
14-3-3γ
S40
PP2AD
B56δ
B56δ
P
14-3-3
S296
S317
S317
PP2AD
CHK1
P
CHK1
CHK1
S317
P
P
P
P
P
S345
S345
S280
S280
S280
P
P
P
PP2AD
B56γ3
NDS
?
B56γ3
ATR
CHK1
UV light Replication stress
P
S345
ATM
S301
CHK1
P
CHK1
S280
S345
S286
Ionizing radiation
P
P
P
S. Fig. 1 (van Tellingen)
Ub
Ub
S301
S301
Ub
P
P
CHK1
CHK1
CHK1
Degradation
S286
S286
S345
P
S345
P
P
P
S280
P
CDS
Growth signal
p90 RSK
FBX6
CHK1
?
