# Supplementary material for: The G2 checkpoint—a node‐based molecular switch
Source: FEBS Open Bio. 2017 Mar 4;7(4):439–55. doi: 10.1002/2211-5463.12206 (PMC5377395; doi:10.1002/2211-5463.12206)
Supplement: Supplementary file 3 [file FEB4-7-439-s003.docx]

Video S1: The G2 checkpoint - a node-based molecular switch. http://onlinelibrary.wiley.com/doi/10.1002/2211-5463.12206/full
